# Utilizing Hirshfeld surface calculations, non-covalent inter­action (NCI) plots and the calculation of inter­action energies in the analysis of mol­ecular packing

**DOI:** 10.1107/S2056989019001129

**Published:** 2019-02-05

**Authors:** Sang Loon Tan, Mukesh M. Jotani, Edward R. T. Tiekink

**Affiliations:** a Research Centre for Crystalline Materials, School of Science and Technology, Sunway University, 47500 Bandar Sunway, Selangor Darul Ehsan, Malaysia; b Department of Physics, Bhavan’s Sheth R. A. College of Science, Ahmedabad, Gujarat 380001, India

**Keywords:** Hirshfeld surface calculations, non-covalent inter­action plots, inter­action energies, mol­ecular packing

## Abstract

The analysis of atom-to-atom and/or residue-to-residue contacts remains a favoured mode of analysing the mol­ecular packing in crystals. In this contribution, additional tools are highlighted as methods for analysis in order to complement the ‘crystallographer’s tool’, *PLATON* [Spek (2009). *Acta Cryst*. D**65**, 148–155]. Thus, a brief outline of the procedures and what can be learned by using *Crystal Explorer* [Spackman & Jayatilaka (2009). *CrystEngComm* **11**, 19–23] is presented. Attention is then directed towards evaluating the nature, *i.e*. attractive/weakly attractive/repulsive, of specific contacts employing *NCIPLOT* [Johnson *et al.* (2010). *J. Am. Chem. Soc*. **132**, 6498–6506]. This is complemented by a discussion of the calculation of energy frameworks utilizing the latest version of *Crystal Explorer*. All the mentioned programs are free of charge and straightforward to use. More importantly, they complement each other to give a more complete picture of how mol­ecules assemble in mol­ecular crystals.

## Abstract 2

The analysis of atom-to-atom and/or residue-to-residue contacts remains a favoured mode of analysing the mol­ecular packing in crystals. This contribution highlights tools for this analysis such as *Crystal Explorer* and *NCIPLOT*, which is used to evaluate the nature, *i.e*. attractive/weakly attractive/repulsive, of specific contacts. This is complemented by a discussion of the calculation of energy frameworks utilizing the latest version of *Crystal Explorer*. These programs are free of charge and straightforward to use. They complement each other to give a more complete picture of how mol­ecules assemble in mol­ecular crystals.

## Introduction   

A widely employed approach to describe the packing of mol­ecular compounds in their crystals is based on describing specific atom-to-atom contacts, such as in conventional *A*—H⋯*D* hydrogen bonding. This analysis is often extended into the highly popular supra­molecular synthon approach (Desiraju, 1995 ▸), whereby residue-to-residue contacts are evaluated as exemplified in the familiar eight-membered carb­oxy­lic acid synthon, *i.e*. {⋯HOCO}_2_. Often sharing the directionality, robustness and utility in mol­ecular packing that characterizes hydrogen bonding is the very well documented phenomenon of halogen bonding (Cavallo *et al.*, 2016 ▸). The electrostatic attraction between ostensibly two (partially) negatively charged entities in halogen bonding is ascribed to an anisotropic distribution of electron density around the halogen atom (*X*) in that at the tip of the C—*X* bond, there is an electron-deficient region, a so-called polar cap or σ-hole (Brinck *et al.*, 1992 ▸; Murray *et al.*, 2007 ▸); π-hole inter­actions rely on a similar concept (Bauzá *et al.*, 2015 ▸). Such σ-hole considerations are now employed to rationalize (Kolář & Hobza, 2016 ▸) the very long-documented secondary bonding inter­actions (Alcock, 1972 ▸; Haiduc, 1997 ▸), more recently repackaged in terms of the participating atoms, *e.g*. tetrel bonding for inter­actions involving Group 14 elements (Bauzá *et al.*, 2013 ▸), pnictogen (Group 15; Scheiner, 2013 ▸), chalcogen (Group 16; Wang *et al.*, 2009 ▸) and even aerogen bonding, *i.e*. inter­actions involving noble gases (Bauzá & Frontera, 2015 ▸), but see Edwards *et al.* (2017 ▸). In mol­ecular crystals of organic mol­ecules, π-systems are well known to participate in identifiable points of contact between mol­ecules by π–π stacking inter­actions (Janiak, 2000 ▸) and C—H⋯π contacts (Nishio, 2004 ▸). In the realm of coordination chemistry, chelate rings can also have π-character (Masui, 2001 ▸) and participate in analogous π–π inter­actions, where one or both of the inter­acting rings is a chelate ring (Malenov *et al.*, 2017 ▸), and C—H⋯π(chelate) inter­actions (Sredojević *et al.*, 2007 ▸; Tiekink & Zukerman-Schpector, 2011 ▸). Metals themselves can associate in the solid state as most famously demonstrated by gold which, owing to relativistic effects, has a significant propensity to form Au⋯Au (aurophilic) inter­actions that provide comparable energies of stabilization to their crystals as do conventional hydrogen-bonding inter­actions and, indeed, can be competitive with these (Schmidbaur, 2000 ▸; Schmidbaur & Schier, 2008 ▸; Tiekink, 2014 ▸). Gold can also form Au⋯π(arene) inter­actions in their crystals (Caracelli *et al.*, 2013 ▸), as do many elements, including main-group elements in low oxidation states (Caracelli *et al.*, 2016 ▸). In the case of the latter, it is the lone-pair of electrons on the heavy element that inter­acts with the π-system, a phenomenon that arises owing to the σ-hole present at the tip of the lone-pair of electrons, analogous to that discussed above. Far from being mere curiosities, inter­actions involving metals, chelate rings and secondary bonding inter­actions impart stabilization energies to mol­ecular packing akin to conventional hydrogen bonding (Tiekink, 2017 ▸).

Confronted by a myriad of different types of identifiable points of contact between ions and mol­ecules in their crystals – the list of inter­actions cited above is not exhaustive and further types are sure to be appreciated and documented in the coming years – describing mol­ecular packing in detail can be a challenge. Ton Spek’s program *PLATON* (Spek, 2009 ▸), downloadable free of charge for academic users from http://www.platonsoft.nl/spek/xraysoft/, is an excellent starting point for such an analysis as the geometric parameters characterizing close contacts, including for non-conventional contacts such as element(lone pair)⋯π(arene) and C—H⋯π(chelate ring) contacts, are evident in the output from *PLATON*. All that is required is the final, validated crystallographic information file (CIF). An obvious limitation here is the application of distance criteria to determine the presence of a contact: inter­actions can and do extend beyond sums of van der Waals radii (Boese *et al.*, 2001 ▸; Dance, 2003 ▸). Other tools are also freely available to gain further insight into the way mol­ecules pack in their crystals. Two in particular form the focus of this contribution, namely Hirshfeld surface analysis and non-covalent inter­action plots. As indicated below, these are easy-to-use programs and can provide complementary information useful for the study of mol­ecular packing. The purpose of the present contribution is to highlight the use of *Crystal Explorer* (Spackman & Jayatilaka, 2009 ▸) and *NCIPLOT* (Johnson *et al.*, 2010 ▸) in the analysis of crystals and to provide po­inters to get the most out of these programs. The inter­ested reader is referred to the original cited papers for more detailed information of the theory behind the different approaches described herein.

A caveat: the popularity and importance of identifying atom-to-atom/residue-to-residue contacts notwithstanding, the perennial question facing those trying to understand how and why mol­ecular crystals form is nicely summarized by the ‘*egg causality dilemma’* – what came first, the chicken or the egg? In the present context, are the identified inter­molecular inter­actions responsible for directing the way mol­ecules assemble in crystals or are the identified inter­molecular inter­actions the result of the formation of crystals? Developing this last point further, in a global mol­ecular packing approach, mol­ecules assemble to minimize free space so that protrusions (‘bumps’) in a mol­ecule are accommodated by impressions (‘craters’) of symmetry-related mol­ecules. It is also noted in this context of close packing considerations that around 83% of mol­ecular compounds crystallize in one of six close-packing space groups (Allen, 2002 ▸). In salient comments underscoring the above are the observations by Dunitz & Gavezzotti (2009 ▸) when writing specifically about weak inter­actions involving hydrogen atoms, in effect, that these atoms have to be accommodated somewhere and are unlikely to adopt repulsive configurations; the same ideas apply equally to other inter­molecular inter­actions. Whatever the origin of the inter­molecular inter­actions revealed in crystals, their identification and analysis, especially in a systematic and thorough manner, is surely a worthwhile enterprise.

## Hirshfeld surface analysis and two-dimensional fingerprint plots   

### Preamble   

The analysis of calculated Hirshfeld surfaces has become an invaluable tool for crystallographers and crystal engineers alike as this provides additional insight into weak inter­molecular inter­actions influential in the packing of mol­ecules in crystals. A Hirshfeld surface is defined by the density weight function of the specific mol­ecule of inter­est (*i.e*. the pro-mol­ecule) over the same sum of density of its nearest neighbour (*i.e*. the pro-crystal), thereby resulting in a 0.5 arbitrary units isosurface, which is similar to that of a van der Waals surface but, unlike the latter, takes into consideration neighbouring mol­ecules and hence provides information about inter­molecular inter­actions (McKinnon *et al.*, 2007 ▸; Spackman & Jayatilaka, 2009 ▸). The Hirshfeld surfaces can be mapped with different properties namely, *d*_norm_, electrostatic potential, shape-index and curvedness. These are useful to accumulate additional information on weak inter­molecular inter­actions. *Crystal Explorer 17* (Turner *et al.*, 2017 ▸) may be downloaded from http://crystalexplorer.scb.uwa.edu.au/downloads.html.

The Hirshfeld surfaces mapped over *d*_norm_ utilize the function of normalized distances *d*_e_ and *d*_i_, where *d*_e_ and *d*_i_ are the distances from a given point on the surface to the nearest atom outside and inside, respectively. The blue, white and red colour conventions used for the *d*_norm_-mapped Hirshfeld surfaces recognize the inter­atomic contacts as longer, at van der Waals separations and short inter­atomic contacts, respectively. The views of Hirshfeld surfaces mapped over the electrostatic potential obtained using the computational chemistry package *Tonto* (Jayatilaka & Grimwood, 2003 ▸), integrated into the *Crystal Explorer 17* program, also enables the visualization of the donors and acceptors of inter­molecular inter­actions through blue and red regions around the partici­pating atoms corresponding to positive and negative electrostatic potential on the surface, respectively. Being a powerful quantum chemistry package for wave-function calculation and surface generation, *Tonto* can be used as an alternative to popular quantum chemistry packages, *e.g. Gaussian16* (Frisch *et al.*, 2106 ▸), and is available in *Crystal Explorer 17*. The package uses Hartree–Fock/DFT theory wave-function calculations based on the input CIF.

The useful measures of curvature, namely curvedness and shape-index, introduced by Koendrink (Koenderink, 1990 ▸; Koenderink & Doorn, 1992 ▸), provide further chemical insight into mol­ecular packing. A surface with low curvedness designates a flat region and may be indicative of π–π stacking in the crystal. On the other hand, a Hirshfeld surface with high curvedness is highlighted as dark-blue edges, which is indicative of an absence of π–π stacking. The shape-index is a qualitative measure of shape and is sensitive to subtle changes in surface shape, particularly in a flat region. Two shape indices differing by sign represent complementary ‘bumps and hollows’. The blue bump-shape and shape-index > 1 belongs to the donor, and that representing a red hollow with index < 1 corresponds to the acceptor of an inter­molecular inter­action.

The two-dimensional fingerprint plot derived from a Hirshfeld surface (Spackman & McKinnon, 2002 ▸; McKinnon *et al.*, 2004 ▸) provides a convenient visual summary of the frequency of each combination of *d*_e_ and *d*_i_ across the surface of a mol­ecule. It is a highly useful method to summarize complex information contained in a crystal. The colour of each point corresponding to the relative area of a (*d*_e_, *d*_i_) pair is recognized as the contribution from different inter­atomic contacts: blue, green and red correspond to small, moderate and greatest contributions whereas an uncoloured region indicates no contribution to the Hirshfeld surface. A fingerprint plot delineated into specific inter­atomic contacts contains information related to specific inter­molecular inter­actions.

To conduct the above calculations, one should employ the final validated CIF as the input to *Crystal Explorer 17*; by default, the program will adjust the *X*—H bond lengths to their neutron-derived values. Specific examples of how each of the above can be applied in the analysis of mol­ecular compounds follows.

### Illustrative examples   

**Figure 1:**
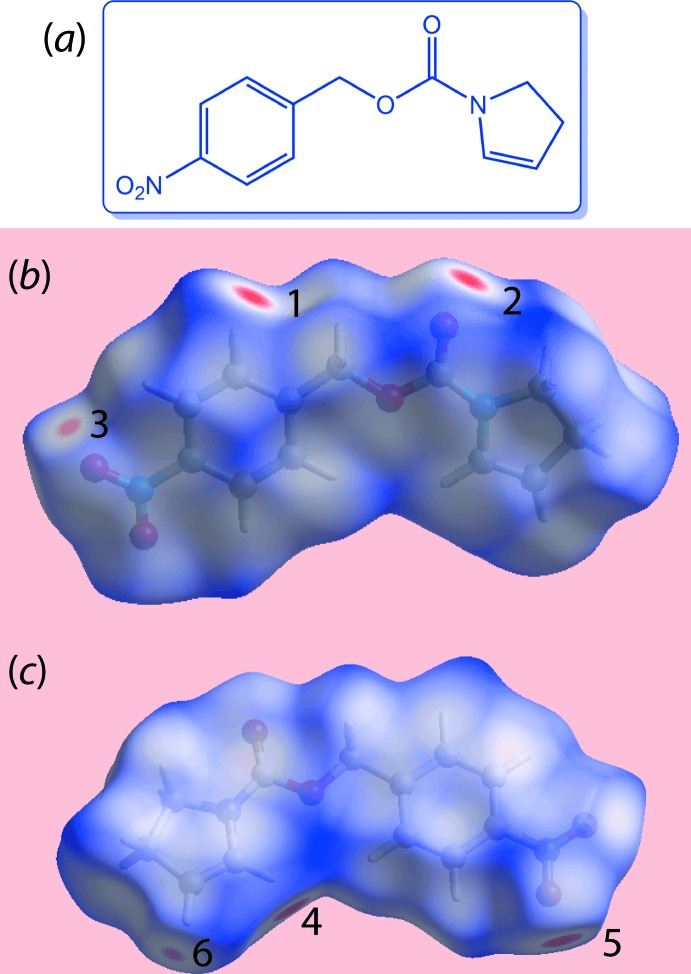
(*a*) Chemical diagram for (I), two views of the Hirshfeld surface mapped over *d*_norm_ for (I) over the ranges (*b*) −0.255 to +1.393 and (*c*) −0.055 to +1.393 arbitrary units; the numbers **4**–**6** indicate points of contact derived from different inter­molecular inter­actions than those indicated in (*b*).

The first example concerns the generation and inter­pretation of Hirshfeld surfaces calculated over *d*_norm_. Fig. 1 ▸(*a*) shows the chemical structure of (4-nitro­phen­yl)methyl 2,3-di­hydro-1*H*-pyrrole-1-carboxyl­ate (C_12_H_10_N_4_O), (I), which was reported recently (Zukerman-Schpector, Soto-Monsalve *et al.*, 2018 ▸). To view the characteristic red spots indicating specific points of contact in the crystal, the Hirshfeld surface mapped over *d*_norm_ was calculated with the default setting of arbitrary units range; rotation of the generated plot enables the identification regions of inter­est, *e.g*. Fig. 1 ▸(*b*) and (*c*). The red spots can be classified as bright, diminutive and faint to correlate (qualitatively) with the strength of inter­molecular contact, *i.e*. as potential hydrogen bonds, weak inter­actions or short inter­atomic contacts.

In Fig. 1 ▸(*b*), the bright-red spots near the hydrogen (indicated with ‘**1**’) and oxygen (‘**2**’) atoms indicate donors and acceptors of a potential C—H⋯O inter­action. The diminutive-red spot near the nitro-oxygen atom (‘**3**’) represents its participation as an acceptor in a comparatively weak C—H⋯O contact (with a pyrrole-hydrogen atom). Additional faint-red spots arising from short inter­atomic contacts can be viewed by reducing the range of arbitrary units in the calculation by modifying the value of the negative arbitrary unit, as is apparent from Fig. 1 ▸(*c*) where additional red regions are highlighted as **4**–**6** (Zukerman-Schpector, Soto-Monsalve *et al.*, 2018 ▸).

**Figure 2:**
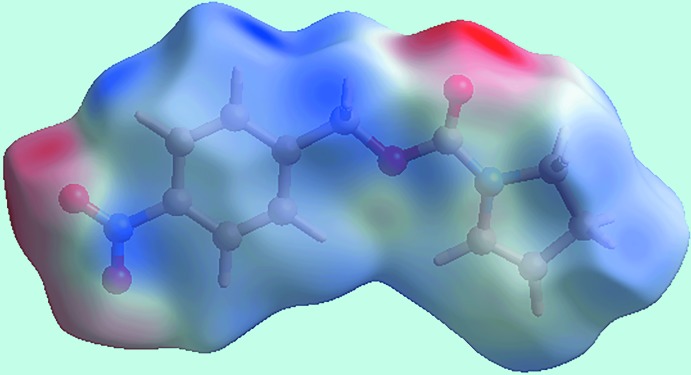
A view of the Hirshfeld surface for (I) mapped over the calculated electrostatic potential in the range −0.077 to +0.056 atomic units (the red and blue regions represent negative and positive electrostatic potentials, respectively).

The Hirshfeld surface mapped over the calculated electrostatic potential for (I) is shown as the image in Fig. 2 ▸. Here, the blue and red regions around the different atoms correspond to positive and negative electrostatic potentials, respectively, *e.g*. red regions are apparent around the oxygen atoms participating in the C—H⋯O contacts mentioned above.

**Figure 3:**
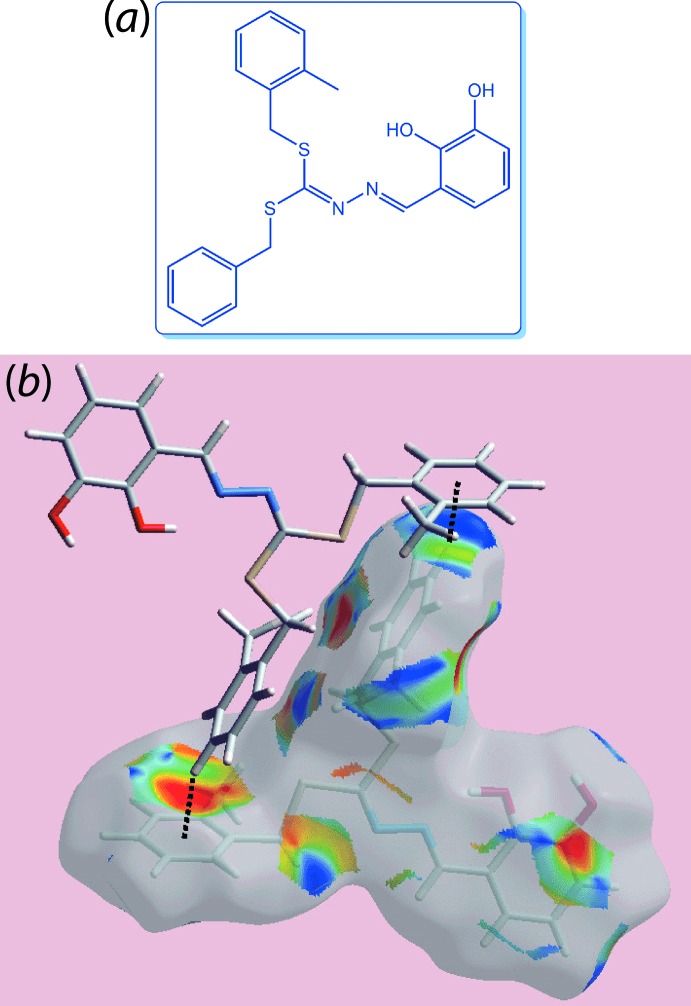
(*a*) Chemical diagram for (II) and (*b*) a view of the Hirshfeld surface mapped with the shape-index property illustrating C—H⋯π/π⋯H—C contacts in the crystal of (II).

The Hirshfeld surfaces mapped with other properties like shape-index and curvedness can be employed to describe the effect of weak inter­molecular inter­actions in a crystal, *e.g*. in the crystal of 3-[(1*Z*)-{2-[bis­({[(2-methyl­phen­yl)meth­yl]sulfan­yl})methyl­idene]hydrazin-1-yl­idene}meth­yl]benzene-1,2-diol (C_23_H_22_N_2_O_2_S_2_) (II), Fig. 3 ▸(*a*) (Yusof *et al.*, 2018 ▸). As an example, the donor and the acceptors of inter­molecular C—H⋯π contacts can be recognized as blue and red regions around the participating atoms on the Hirshfeld surfaces mapped over shape-index properties corresponding to C—H⋯π/π⋯H—C (often abbreviated as C⋯H/H⋯C) contacts, as shown in Fig. 3 ▸(*b*) for (II).

**Figure 4:**
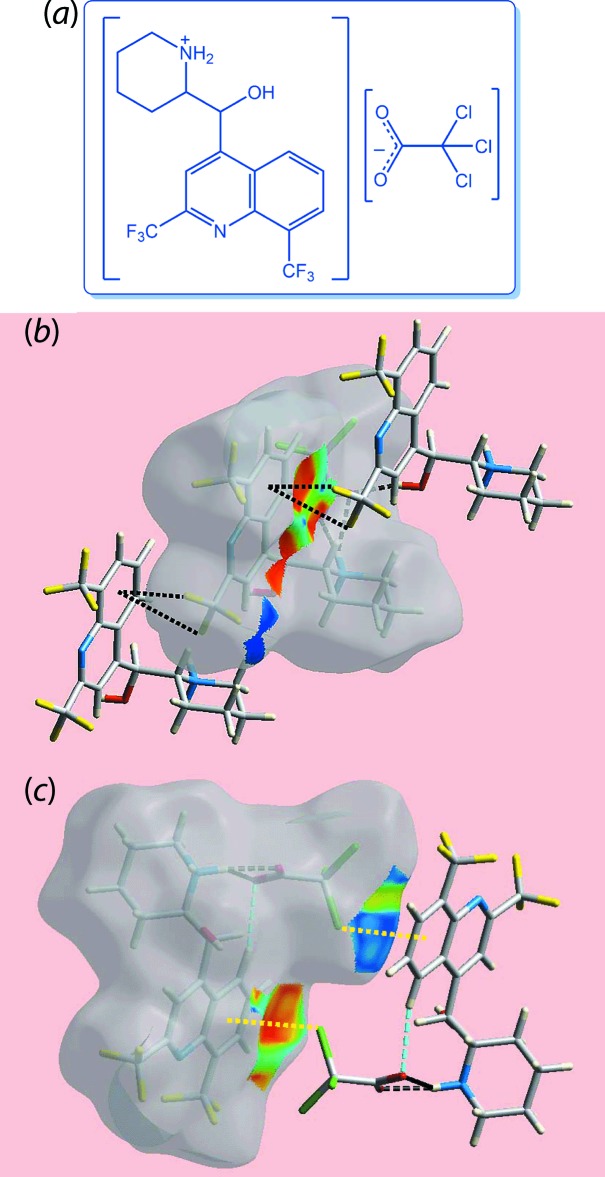
(*a*) Chemical diagram for (III) and views of the Hirshfeld surface mapped with the shape-index property illustrating (*b*) C–F⋯π/π⋯F—C and (*c*) C—Cl⋯π/π⋯Cl—C contacts in the crystal of (III) through black and yellow dashed lines, respectively.

Thus far, the focus has been upon conventional inter­actions such as C—H⋯O and C—H⋯π. It is noted that Hirshfeld surface analysis is equally useful for identifying other, less-common points of contact between mol­ecules. This point is exemplified in the crystal of the halide-rich salt 2-{[2,8-bis(tri­fluoro­meth­yl)quinolin-4-yl](hy­droxy)meth­yl}piperidin-1-ium tri­chloro­acetate (C_19_H_17_N_2_OF_6_^+^·C_2_Cl_3_O_2_^−^) (III), Fig. 4 ▸(*a*) (Wardell *et al.*, 2018 ▸). In (III), C—*X*⋯π contacts involving *X* = F and Cl are evident, and these are readily apparent when the Hirshfeld surface is mapped with the shape-index property as shown in Fig. 4 ▸(*b*) and (*c*).

**Figure 5:**
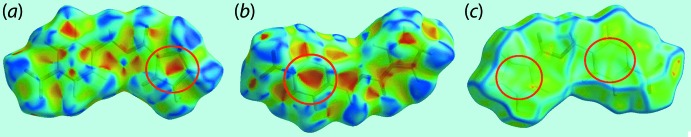
(*a*) Views of the Hirshfeld surface for (I) mapped over the shape-index property highlighting blue regions about bright-red spots within the (*a*) pyrrolyl and (*b*) benzene rings, and (*c*) the Hirshfeld surface mapped over curvedness indicating flat regions around the pyrrolyl and benzene rings. The respective rings are highlighted by the red circles.

To examine the influence of π–π stacking on the mol­ecular packing, an analysis of the Hirshfeld surface mapped over the shape-index and curvedness properties can be instructive. The structure of (I), Fig. 1 ▸(*a*), is used an example as the crystal features π(pyrrole)–π(nitro­benzene) stacking inter­actions (Zukerman-Schpector, Soto-Monsalve *et al.*, 2018 ▸). Two views of the Hirshfeld surface mapped over the shape-index property are shown in Fig. 5 ▸(*a*) and (*b*). From these, the π–π stacking between the rings is indicated by the appearance of small blue regions surrounding bright-red spots within the respective five- and six-membered rings. The presence of π–π stacking is also evident as the flat regions around the pyrrole and benzene rings on the Hirshfeld surface mapped over curvedness, Fig. 5 ▸(*c*).

**Figure 6:**
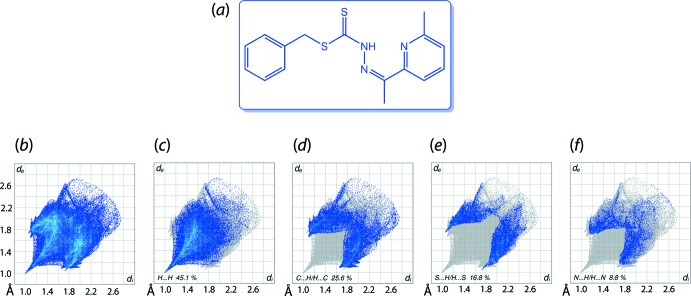
(*a*) Chemical diagram for (IV), (*b*) the full two-dimensional fingerprint plot for (IV) and fingerprint plots delineated into (*c*) H⋯H, (*d*) C⋯H/H⋯C, (*e*) S⋯H/H⋯S and (*f*) N⋯H/H⋯N contacts.

Two-dimensional fingerprint plots can also be used to analyse the calculated Hirshfeld surface of a mol­ecule. Typically, the overall fingerprint plot is calculated, encompassing all inter­molecular contacts, as well as the delineated (or decomposed) fingerprint plots, which focus on specific inter­actions. This is illustrated for the structure of *S*-benzyl 3-[1-(6-methyl­pyridin-2-yl)ethyl­idene]di­thio­carbazate (C_16_H_17_N_3_S_2_) (IV), Fig. 6 ▸(*a*) (Omar *et al.*, 2018 ▸). The overall fingerprint plot for (IV) is shown in Fig. 6 ▸(*b*) and those delineated into H⋯H, C⋯H/H⋯C, S⋯H/H⋯S and N⋯H/H⋯N inter­actions are shown in Fig. 6 ▸(*c*)–(*f*), respectively. While it is likely there are other identifiable points of contact that can be highlighted in the crystal, these may be of limited significance and do not require detailed discussion nor illustration. In the present case of (IV), the relative percentage contributions to the overall Hirshfeld surface are presented in Table 1 ▸. Ideally, in the absence of rounding-up errors, the relative percentage contributions should sum to 100%. Fingerprint plots would normally be presented for the more significant contributions to the surface unless a special feature of the mol­ecular packing deserves highlighting. As seen from Fig. 6 ▸(*b*), the overall two-dimensional fingerprint plot is the sum of the delineated plots, having features drawn from the plots shown in Fig. 6 ▸(*c*)–(*f*). It is usually the case that the main contribution to the overall surface arises from H⋯H contacts. Also noteworthy is that while often forming the focus of discussion, conventional hydrogen bonding often makes relatively small percentage contributions to the overall surface.

When evaluating fingerprint plots, peaks/tips/features occurring at values less than the sum of the van der Waals radii need to be looked for. For example, in the case of H⋯H contacts, Fig. 6 ▸(*c*), the tip occurs at *d*_e_ + *d*_i_ < 2.40 Å, *i.e*. less than 2 × the van der Waals radius of hydrogen, suggestive of some of sort of contact, whether it be attractive or repulsive. The same procedure is followed for all other contacts. In (IV), the forceps-like tips in Fig. 6 ▸(*d*) and (*e*) correspond to inter­actions less than the sum of the respective van der Waals radii but, not so in Fig. 6 ▸(*f*).

Hirshfeld surface analyses are equally useful for assessing multi-component crystals, including solvates, salts and structures with *Z′* > 1. In these situations, not only should the overall fingerprint plots be plotted but also those for the individual components. In a recent study where four cations and four anions comprised the crystallographic unit, distinctive features were evident in the fingerprint plots and in the relative percentage contributions of different inter­actions to the Hirshfeld surfaces for each individual component of the structure, which enabled the confirmation of the space group (Jotani *et al.*, 2019 ▸). The calculation of Hirshfeld surfaces over the electrostatic potential will indicate inter­acting regions of the constituent mol­ecules and can often be a useful starting point for analysis. Less confidence in inter­pretation will be likely in structures featuring disorder.

## Non-covalent inter­action plots   

It is a fair assumption that under ambient conditions mol­ecules, by and large, assemble into crystals optimizing attractive inter­actions while at the same time minimizing repulsive inter­actions. Given the nature and broad range of different inter­molecular inter­actions now widely discussed in the crystallographic literature, it is salient to confirm whether such inter­actions are indeed attractive and therefore, stabilizing. In their landmark paper entitled ‘*Revealing Noncovalent Inter­actions*’, Yang and co-workers (Johnson *et al.*, 2010 ▸; Contreras-García *et al.*, 2011 ▸) put forward a convenient, rapid and user-friendly approach to enable the discrimination between attractive and repulsive inter­actions. The method relies solely on the three-dimensional atomic coordinates and is equally applicable to macromolecular systems. The program *NCIPLOT* may be downloaded, again without charge, from http://www.lct.jussieu.fr/pagesperso/contrera/nciplot.html.

In short, the reduced density gradient is plotted as a function of the density (mapped as isosurfaces) over the mol­ecule of inter­est. The sign of the second Hessian eigenvalue times the electron density [*i.e*. sign(λ^2^)ρ in atomic units] enables the identification of attractive/stabilizing (favourable) or repulsive (unfavourable) inter­actions. The derived results are readily visualized employing the VMD (visual mol­ecular dynamics) mol­ecular graphics viewer (Humphrey *et al.*, 1996 ▸), which is freely available from https://www.ks.uiuc.edu/Research/vmd/.

The nature of the specific inter­actions is highlighted through a red–blue–green colour scheme on the calculated isosurface. A strong attractive inter­action is indicated in blue whereas red indicates a strong repulsive inter­action. Weak inter­actions are highlighted by a green isosurface.

**Figure 7:**
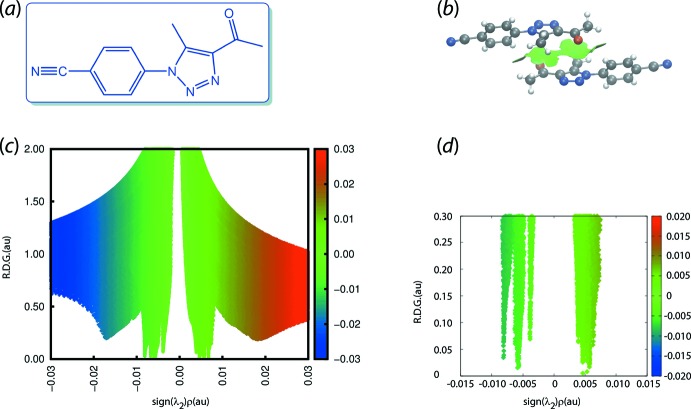
(*a*) Chemical diagram for (V), (*b*) non-covalent inter­action plot of the two-mol­ecule aggregate (centrosymmetric) sustained by carbonyl-C= O⋯π(triazol­yl) inter­actions, (*c*) a plot of the reduced density gradient *versus* the electron density multiplied by the sign of the second Hessian eigenvalue and (*d*) detail of (*c*) highlighting the weakly attractive nature of the carbonyl-C=O⋯π(triazol­yl) inter­action.

A recent example employing this approach is illustrated for the structure of 4-(4-acetyl-5-methyl-1*H*-1,2,3-triazol-1-yl)benzo­nitrile (V), Fig. 7 ▸(*a*) (Zukerman-Schpector, Dias *et al.*, 2018 ▸). One identified contact between centrosymmetrically related mol­ecules is a carbonyl-C=O⋯π(triazol­yl) inter­action, where the inter­acting species are approximately parallel. As seen in the images of Fig. 7 ▸(*b*), a green isosurface is evident between the participating residues suggesting the inter­action is weakly attractive. Fig. 7 ▸(*c*) shows an overall plot of the reduced density gradient *versus* the electron density times the sign of the second Hessian eigenvalue. Fig. 7 ▸(*d*) is an expanded version of Fig. 7 ▸(*c*) highlighting the weakly attractive nature of carbonyl-C=O⋯π(triazol­yl) inter­action in the negative region of the plot.

## Inter­action energies and energy frameworks   

A new feature has been recently incorporated into *Crystal Explorer 17* to enable the calculation of pair-wise inter­action energies within a crystal by summing up four energy components comprising electrostatic (*E*_ele_), polarization (*E*_pol_), dispersion (*E*_dis_) and exchange-repulsion (*E*_rep_) (Turner *et al.*, 2015 ▸). Users may apply two energy models available in the software to perform the calculation, *i.e*. CE-B3LYP/6-31G(*d,p*) and CE-HF/3-21G, which have been appropriately scaled to reproduce the B3LYP-D2/6-31G(*d,p*) counterpoise-corrected energies with a small mean absolute deviation of 2.4 and 4.7 kJ mol^−1^ for the respective models based on a set of crystal structures covering neutral organic mol­ecules, organic salts, solvates, coordination compounds and radicals (Turner *et al.*, 2015 ▸; Mackenzie *et al.*, 2017 ▸).

**Figure 8:**
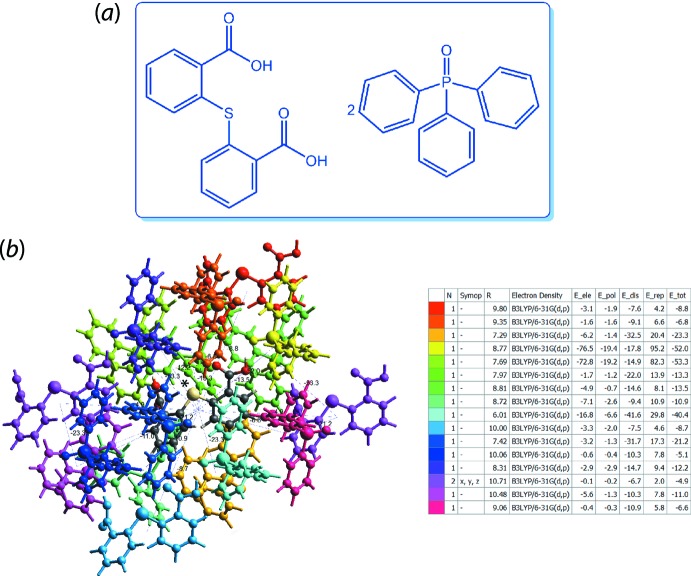
(*a*) Chemical diagram for (VI) and (*b*) the colour-coded inter­action mapping within 3.8 Å of the centring S1 (marked by an asterisk) mol­ecular cluster.

The calculation of inter­action energies is generally straightforward for crystal structures with *Z′* = 1, whereby users simply need to generate a cluster of mol­ecules within a radius of 3.8 Å (*i.e*. the default value for mol­ecules comprising light atoms) for a selected reference mol­ecule and subsequently subject it to energy calculation upon setting the relevant parameters such mol­ecular charge, multiplicity and energy model. For multi-component crystals or crystals with *Z′* > 1, the wave-functions for each unique mol­ecule need to be calculated prior to obtaining the inter­action energies for a cluster of mol­ecules, as this is to ensure that the terrain of energy will encompass all pair-wise energies between the unique mol­ecules, be they hetero-mol­ecules (*A*⋯*B*) or within homo-mol­ecules (*A*⋯*A*′ or *B*⋯*B′*), *etc*. An example of such a calculation was demonstrated in a recent study on the co-crystal comprising two mol­ecules of 2,2′-thiodi­benzoic acid (S1 and S2) and four mol­ecules of tri­phenyl­phosphane oxide (P1, P2, P3 and P4) in the asymmetric unit, (VI), Fig. 8 ▸(*a*) (Tan & Tiekink, 2018 ▸). Here, the pair-wise energy was first obtained for the respective pairs of inter­acting mol­ecules (*i.e*. S1⋯P1, S1⋯P4, S2⋯P3 and S2⋯P4) prior to the calculation of inter­action energies within 3.8 Å for the S1 and S2 clusters.

Useful information can be obtained upon the successful calculation of inter­action energies. For instance, the calculation results in a colour-coded mol­ecular cluster related to the specific inter­action energy, Fig. 8 ▸(*b*). The individual energy components (*E*_ele_, *E*_pol_, *E*_dis_ and *E*_rep_) as well as the sum of energy components (*E*_tot_) for the inter­actions relative to the reference mol­ecule (based on the colour scheme) are provided in the accompanying table under the information dialogue; the individual energy components are not scaled but the *E*_tot_ is scaled according to the relevant energy model (Mackenzie *et al.*, 2017 ▸). Apart from these energy data, other information can be obtained from the generated table, such as the existence of rotational symmetry operations with respect to the reference mol­ecule (*Symop*), the centroid-to-centroid distance between the reference mol­ecule and inter­acting mol­ecules (*R*), as well as the number of pair(s) of inter­acting mol­ecules with respect to the reference mol­ecule (*N*), which is useful in calculating a lattice energy of a crystal.

**Figure 9:**
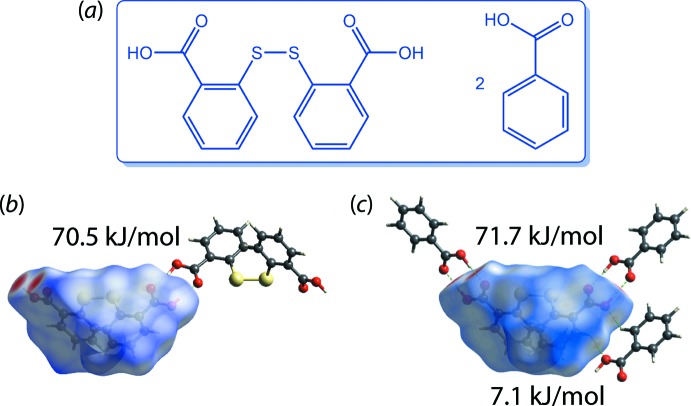
(*a*) Chemical diagram for (VII) and qu­anti­fication of the strength of specific inter­actions through energy calculation that correlates with the *d*_norm_ mapping on the pro-mol­ecule surface for the (*b*) 2,2′-di­thiodi­benzoic acid and (*c*) 1:2 co-crystal of 2,2′-di­thiodi­benzoic acid and benzoic acid.

As mentioned in the *Hirshfeld surface analysis and two-dimensional fingerprint plots* section, Hirshfeld surface analysis is used to identify any close contacts present in a crystal through mapping of *d*_norm_ on the pro-mol­ecule surface, and the strength of the close contacts may be estimated qualitatively through the intensity of the red spots observed on the surface or *via* the *d*_i_ + *d*_e_ contact distance as determined from a delineated fingerprint plot. With the availability of an immensely useful feature in the newly released *Crystal Explorer 17*, users may now qu­antify the strength of contacts by calculating the inter­action energies and correlate this information with the results of the Hirshfeld surface analysis. This feature is especially useful in crystal engineering, for which it can be applied to compare and subsequently fine-tune the strength of inter­actions for any closely related analogues in designing structures with specific inter­actions for desirable applications. This idea is illustrated in a recent study of the 2:1 co-crystal formed between 2,2′-di­thiodi­benzoic acid and benzoic acid (C_14_H_10_O_2_S_2_·C_7_H_6_O_2_), (VII), Fig. 9 ▸(*a*) (Tan & Tiekink, 2019 ▸). The inter­actions between the carb­oxy­lic acid residues *via* {⋯HOC=O}_2_ synthons in 2,2′-di­thiodi­benzoic acid (Humphrey & Wood, 2003 ▸), Fig. 9 ▸(*b*), are the same as in the structure of this conformer in the 1:2 co-crystal with benzoic acid, and about 10 × greater than a benzene-C—H⋯O(hydrox­yl) inter­action, Fig. 9 ▸(*c*).

An option also exists in the new version of the *Crystal Explorer 17* software to simulate energy frameworks, *i.e*. a graphical representation of the individual energy components depicted as cylinders joining the centroids of inter­acting mol­ecular pairs, in which *E*_ele_, *E*_dis_ and *E*_tot_ are, respectively, colour-coded in red, green and blue, and with the radius of the corresponding cylinders proportional to the magnitude of inter­action energy (Turner *et al.*, 2015 ▸).

**Figure 10:**
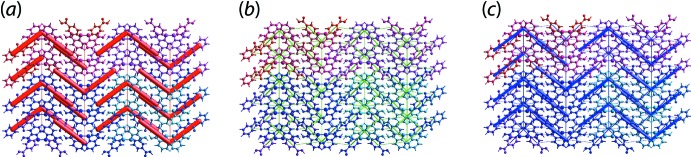
Energy frameworks calculated for (VII) viewed along the *a*-axis direction, showing the (*a*) electrostatic potential force, (*b*) dispersion force and (*c*) total energy diagrams. The cylindrical radii are proportional to the relative strength of the corresponding energies and they were adjusted to the same scale factor of 50 with a cut-off value of 5 kJ mol^−1^ within 4 × 4 × 4 unit cells.

The simulation of the energy framework is an extended feature established based on the calculation of inter­action energies. To simulate a framework, users first need to obtain the wave-functions for all unique pairs of inter­acting mol­ecules as described earlier. Subsequently, a cluster of mol­ecules within an appropriate number of unit cells needs to be generated depending on the completeness of the framework, *e.g*. a cluster of mol­ecules within 2 × 2 × 2 unit cells may be a good start. Upon the completion of the energy calculations for the mol­ecular cluster within the unit cells, the frameworks can be obtained through manifestation of the corresponding cylinder rods; these may need to be adjusted by a scale factor for direct comparison. An appropriate energy threshold can be set to omit any weak inter­actions for purposes of clarity. An illustrative example is given in Fig. 10 ▸ for the structure of (VII) (Tan & Tiekink, 2019 ▸).

**Figure 11:**
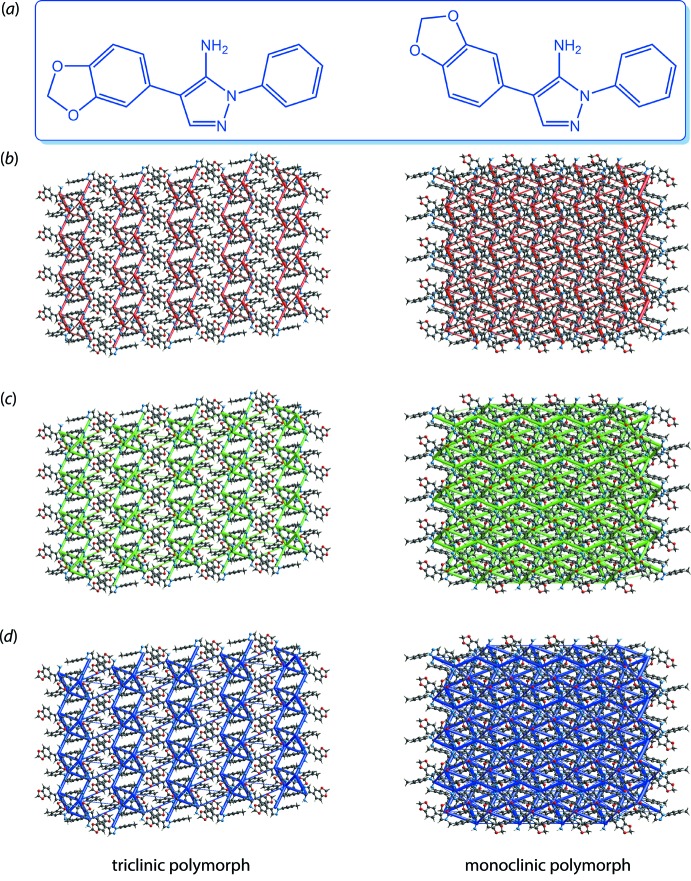
(*a*) Chemical diagrams for the conformational polymorphs of (VIII) – the triclinic form comprises one of each conformation while the monoclinic form displays only the conformation shown on the right-hand side. A comparison of the energy frameworks composed of (*b*) electrostatic potential force, (*c*) dispersion force and (*d*) total energy for the triclinic and monoclinic polymorphs. The energy frameworks were adjusted to the same scale factor of 80 with a cut-off value of 9 kJ mol^−1^ within 4 × 4 × 4 unit cells.

The calculation of energy frameworks was developed to better understand the topology of the overall inter­action energies between the constituents of a crystal. For example, such an approach has found application in rationalization of the mechanical behaviour of drugs with relation to their tabletability (the ease of forming a tablet from a powder) (Turner *et al.*, 2015 ▸). The importance of this functionality can be clearly seen when it is applied to polymorphs, as it allows users to directly compare the topological differences of the energy components between the structures, and potentially enable the correlation of energy frameworks with the physicochemical properties or packing behaviour of the polymorph of inter­est. As an example, the calculated energy frameworks for two conformational polymorphs of 4-(2*H*-1,3-benzodioxol-5-yl)-1-(4-methyl­phen­yl)-1*H*-pyrazol-5-amine (C_15_H_13_N_3_O_2_) (VIII), Fig. 11 ▸(*a*) (Gajera *et al.*, 2013 ▸; Jotani *et al.*, 2015 ▸) is described. One polymorph is triclinic with *Z′* = 2 (Gajera *et al.*, 2013 ▸) while the other is monoclinic with *Z′* = 1 (Jotani *et al.*, 2015 ▸). The main difference between the two polymorphs is that one of the independent mol­ecules in the triclinic form adopts a *syn* disposition for the dioxolyl fused-ring system with respect to the amino substituent connected to the central pyrazolyl ring but the other adopts an *anti*-arrangement (Gajera *et al.*, 2013 ▸). In the monoclinic form, the mol­ecules appear entirely in the *syn* form (Jotani *et al.*, 2015 ▸). Through a powder X-ray diffraction study, it was found that the *syn*- and *anti*-orientations exist in 3:1 ratio (Jotani *et al.*, 2015 ▸). This result is affirmed by a study of the energy frameworks for the polymorphs in which the monoclinic form exhibits a more compact framework in comparison to the triclinic form, as evidenced by the relatively thicker cylindrical radius at the same scale factor, which gives an indication that greater stabilization energies exist in the monoclinic system, Fig. 11 ▸(*b*)–(*d*).

## Conclusion   

The ready availability and ease of use of *Crystal Explorer 17*, including the calculation of energy frameworks, and *NCIPLOT* suggests these should be routinely employed tools in describing the mol­ecular packing, as they complement the geometric analysis provided by the indispensable tool, *PLATON*. In short, utilizing these additional tools will ensure that the practitioner will get the most out of their experiments.

## Figures and Tables

**Table 1:** Relative percentage contributions of close contacts to the Hirshfeld surface of (IV)

Contact	Percentage contribution
H⋯H	45.1
C⋯H/H⋯C	25.6
S⋯H/H⋯S	16.8
N⋯H/H⋯N	8.8
C⋯S/S⋯C	2.1
S⋯N/N⋯S	0.9
C⋯C	0.7
